# Neoadjuvant chemotherapy and immunotherapy followed by radical radiotherapy for locally advanced cervical cancer: efficacy and safety

**DOI:** 10.3389/fimmu.2026.1784958

**Published:** 2026-06-22

**Authors:** Qianxue Wei, Hui Qu, Yatong Yu, Song Gao, Yuxin Jia, Yu Xia, Xiuqin Li, Wen Gu

**Affiliations:** 1 Department of Obstetrics and Gynecology, Shengjing Hospital of China Medical University, Shenyang, China; 2 Ultrasound Department, Shengjing Hospital of China Medical University, Shenyang, China; 3 Department of Oncology, Shengjing Hospital of China Medical University, Shenyang, China

**Keywords:** efficacy, intestinal toxicity, locally advanced cervical cancer, neoadjuvant chemoimmunotherapy, radical radiotherapy, safety

## Abstract

**Objective:**

This study aimed to evaluate the short-term efficacy and safety of neoadjuvant chemoimmunotherapy (NACI) followed by radical radiotherapy (RR) in patients with locally advanced cervical cancer (LACC).

**Methods:**

A retrospective analysis was conducted on 60 patients with FIGO 2018 stage IIIA-IVA cervical cancer between June 2022 and July 2024. The experimental group (n=30) received 1–3 cycles of NACI (anti-PD-1/PD-L1 therapy plus platinum-based chemotherapy) followed by RR, which was administered either alone or concurrently with immunotherapy/chemotherapy. The control group (n=30) received RR with or without concurrent chemotherapy. Treatment response was assessed using RECIST 1.1 incorporating gynecological examination.

**Results:**

After NACI, the objective response rate (ORR) was 93.3% in the experimental group, increasing to 100% post-radiotherapy. The complete response (CR) rate was comparable between groups (90.0% vs. 86.7%, P = 1.000). With a median follow-up of 20.5 months, the 2-year progression-free survival (PFS) was significantly higher in the experimental group (82.5% vs. 66.0%; HR = 0.364, P = 0.0303), though overall survival (OS) did not differ significantly. The experimental group had a significantly lower incidence of radiotherapy-related intestinal toxicity (20% vs. 60%, P = 0.0033). All immune-related adverse events were grade 1-2.

**Conclusion:**

For high-risk LACC, NACI followed by RR was associated with a significantly lower incidence of radiotherapy-related intestinal toxicity. A potential PFS benefit was also observed, although its independent effect could not be confirmed in this limited sample. These findings warrant further investigation in larger prospective trials.

## 1 Background

Cervical cancer remains the fourth most common malignancy in women worldwide in both incidence and mortality, with an estimated 660,000 new cases and 350,000 deaths in 2022 (1–3). At initial diagnosis, approximately 37% of patients present with locally advanced disease, defined as stages IB3-IIA2 to IVA under the FIGO 2018 staging system. Although concurrent chemoradiotherapy (CCRT) remains the standard of care for locally advanced cervical cancer (LACC), nearly 30% of patients still experience recurrence or death within five years. A 2013 large retrospective study (4) of 10,012 cervical cancer patients in China revealed a five-year survival rate of less than 60% for those with FIGO stage III-IV disease. Furthermore, the phase III OUTBACK trial (5) demonstrated that the addition of adjuvant chemotherapy to CCRT not only failed to improve survival outcomes but also significantly increased acute treatment-related toxicity.

In recent years, immune checkpoint inhibitors (ICI) have shown promising efficacy across multiple solid tumors, providing new therapeutic avenues for cervical cancer. The PACS trial (6) led by Professor Liu Jihong’s team and the NACI study (7, 8) directed by Professor Li Kezhen’s group both reported that neoadjuvant chemoimmunotherapy (NACI) in patients with IB3–IIA2 stage disease effectively reduces tumor burden and significantly improves rates of R0 resection and pathological complete response (pCR) following radical hysterectomy. Nevertheless, the efficacy and safety of this approach remain unexplored in the more aggressive and often surgically unresectable IIB-IVA patient population. As summarized in a recent comprehensive review (9), treatment advances across the cervical cancer spectrum—including immune−checkpoint inhibitors and neoadjuvant/induction chemotherapy—have reduced morbidity and improved survival, yet high−risk locally advanced disease remains challenging.

This study was designed to explore a potential treatment strategy. We performed a retrospective analysis using real-world data from patients diagnosed with locally advanced cervical cancer who were treated with the “neoadjuvant chemoimmunotherapy (NACI) followed by radical radiotherapy (RR)” regimen at Shengjing Hospital of China Medical University between June 2022 and July 2024. The analysis focused on patients with stage IIIA-IVA disease (FIGO 2018), a subgroup generally associated with high tumor burden and less favorable prognosis, with the aim of generating preliminary evidence that may inform future therapeutic approaches for this population.

## 2 Methods

### 2.1 Study cohort

This retrospective study included 60 patients with locally advanced cervical cancer who were treated between June 2022 and July 2024. Based on the treatment modality, patients were categorized into two groups: those who received neoadjuvant chemoimmunotherapy followed by radical radiotherapy (the experimental group, N = 30) and those who received radical radiotherapy with or without concurrent chemotherapy (the control group, N = 30). To minimize selection bias, propensity score matching was employed based on key clinical characteristics including age, FIGO (2018) stage, and tumor size. (all P > 0.05) (**Table 1**).

**Table 1:** Clinical characteristics of the study groups (N=60).

Characteristic	Experimental group N=30 (%)	Control group N=30 (%)	P-value
Median age (range)	59.5 (36-73)	58 (35-72)	
FIGO stage (2018)			
IIIA	1	1	0.6834
IIIB	8	8	
IIIC1-r	13	13	
IIIC2-r	6	8	
IVA	2	0	
Median tumor diameter, cm (range)	6.65 (3.5-11.0)	6.0 (3.5-9.0)	
Site of Metastasis			
Pelvic lymph nodes	16	20	0.2957
Retroperitoneal lymph nodes	6	8	
Bladder or rectum	2	0	
Pathological Type			
Squamous cell carcinoma	29	29	1.0000
Adenocarcinoma	1	1	
Number of NACI Cycles			
1	5	NA	
2	20	NA	
3	5	NA	
Concurrent Treatment During Radiotherapy			
Concurrent immunotherapy	5	NA	
Concurrent chemotherapy	11	28	
Concurrent chemoimmunotherapy	10	NA	
None	4	2	
Treatment After Radiotherapy			
Maintenance immunotherapy	9	NA	
Chemotherapy	0	5	
Chemoimmunotherapy	2	0	
None	19	25	

NACI, neoadjuvant chemoimmunotherapy; RR, radical radiotherapy; NA, Not Applicable - Control group did not receive NACI treatment.

### 2.2 Data collection, handling of missing data, and blinding for efficacy assessment

Clinical data were retrospectively collected from the electronic medical record system of Shengjing Hospital of China Medical University. Baseline characteristics, treatment details, adverse events, and follow-up information were extracted using a standardized case report form by two independent research assistants. Any discrepancies were resolved by discussion with a senior investigator.

For missing data, we first verified the original medical records. Variables with missingness >10% were not included as predictors. For the few variables with sporadic missing values (all with missing rate <5%), multiple imputation was performed using the fully conditional specification method with five imputed datasets; sensitivity analyses using complete−case analysis yielded similar results, indicating that the impact of missing data was minimal. Variables with 5%-10% missingness were handled by sensitivity analysis using both imputation and complete-case analysis.

To minimize recall and evaluation bias, the assessment of treatment response (RECIST 1.1) and toxicity (CTCAE v5.0) was performed by two independent gynecologic oncologists who were blinded to the treatment allocation (experimental vs. control). The blinding was maintained by removing all treatment-related information from the imaging files and clinical notes before evaluation. A third independent reviewer was consulted in case of disagreement. The radiological review (magnetic resonance imaging) was conducted by a radiologist who was also blinded to group assignment. This prospective blinding strategy for retrospective data was feasible because all response evaluations were performed after the completion of the study follow-up, using anonymized and randomly ordered patient records.

### 2.3 Treatment protocol and efficacy evaluation

All 30 patients in the experimental group received neoadjuvant chemoimmunotherapy (NACI). The treatment regimen in the experimental group was as follows: patients received 1 to 3 cycles of neoadjuvant chemoimmunotherapy (NACI), with the specific number of cycles determined by clinical response and tolerance. The NACI regimen consisted of a platinum-based doublet chemotherapy (TP or TC) combined with an immune checkpoint inhibitor (tislelizumab, sintilimab, or cadonilimab). Following induction therapy, all patients underwent radical radiotherapy (RR), which included intensity-modulated radiation therapy (IMRT) and image-guided adaptive brachytherapy (IGABT), with a total dose to point A ≥ 85 Gy (EQD_2_). During radiotherapy, concurrent immunotherapy, weekly cisplatin (40 mg/m²), or a combination of both was permitted. Concurrent and maintenance therapies are detailed in **Table 1**.

In the control group, all patients received radical radiotherapy. Among them, 28 patients (93.3%) received concurrent chemotherapy, while 2 did not (as detailed in **Table 1**). Following radiotherapy, they could opt for 2–3 cycles of consolidation systemic chemotherapy based on their preference and tolerance.

Tumor response was assessed according to RECIST 1.1 criteria based on gynecological examination and imaging. For the primary comparison of objective response rate (ORR), the assessment conducted at 4 weeks after completion of all protocol-defined radiotherapy (i.e., after NACI+RR in the experimental group and after RR in the control group) was used. Patients in the experimental group underwent an additional response assessment after NACI and before radiotherapy for exploratory analysis.

The primary endpoint of this study was progression-free survival (PFS). Secondary endpoints included objective response rate (ORR) assessed 4 weeks after radiotherapy, overall survival (OS), incidence of immune-related adverse events (irAEs), and radiotherapy-related adverse events.

### 2.4 Safety

Treatment-related adverse events were monitored and graded throughout the study period according to the Common Terminology Criteria for Adverse Events (CTCAE) version 5.0. For the diagnosis and management of immune-related adverse events (irAEs), we referenced the CSCO Guidelines for the Management of Toxicity Related to Immune Checkpoint Inhibitors. Radiotherapy-related toxicities, particularly those affecting the intestinal and urinary systems, were specifically documented.

### 2.5 Ethical considerations and informed consent

This retrospective study was conducted in accordance with the Declaration of Helsinki and was approved by the Ethics Committee of Shengjing Hospital of China Medical University. Given that the research involved the analysis of pre-existing, de-identified clinical data and posed no more than minimal risk to the participants, the Ethics Committee granted a waiver of the requirement for informed consent. This waiver is in compliance with applicable national regulations and institutional policies for retrospective studies of this nature.

### 2.6 Subgroup definitions and multivariate analysis

To address potential confounding from heterogeneous concurrent treatment regimens during radical radiotherapy (RR), we adjusted for the specific therapy administered concurrently with RR by including it as a covariate in a multivariate Cox regression model. In the experimental group (NACI followed by RR), patients were categorized into four subgroups: (1) RR alone, (2) RR with concurrent immunotherapy (without chemotherapy), (3) RR with concurrent chemotherapy (without immunotherapy), and (4) RR with concurrent chemoimmunotherapy (both). In the control group (RR with or without concurrent chemotherapy), patients were divided into: (1) RR alone, and (2) RR with concurrent chemotherapy. To isolate the independent effect of neoadjuvant chemoimmunotherapy (NACI) on progression-free survival (PFS), a multivariate Cox proportional hazards regression model was performed. The model included the following covariates: treatment group (experimental vs. control) and concurrent treatment during RR (categorized as above). Hazard ratios (HRs) with 95% confidence intervals (CIs) were calculated. A two-sided P < 0.05 was considered statistically significant.

### 2.7 Statistical analysis

All statistical analyses were performed using GraphPad Prism (version 10.0.2 for Windows; GraphPad Software, Inc., USA). A two-sided P-value of less than 0.05 was considered statistically significant. Normally distributed continuous variables are presented as mean ± standard deviation (mean ± SD) and were compared using the independent samples t-test. Non-normally distributed continuous data are expressed as median and interquartile range (median [IQR]) and compared with the Mann−Whitney U test. Categorical variables are summarized as frequency and percentage [n (%)] and were compared between groups using the χ² test or Fisher’s exact test, as appropriate. Ordinal data were analyzed using the Wilcoxon rank-sum test. Survival curves for overall survival (OS) and progression-free survival (PFS) were generated by the Kaplan–Meier method. Group differences in survival were compared using the log-rank test.

## 3 Results

### 3.1 Short-term efficacy evaluation

#### 3.1.1 Tumor response after neoadjuvant chemoimmunotherapy in the experimental group

**Figure 1:**
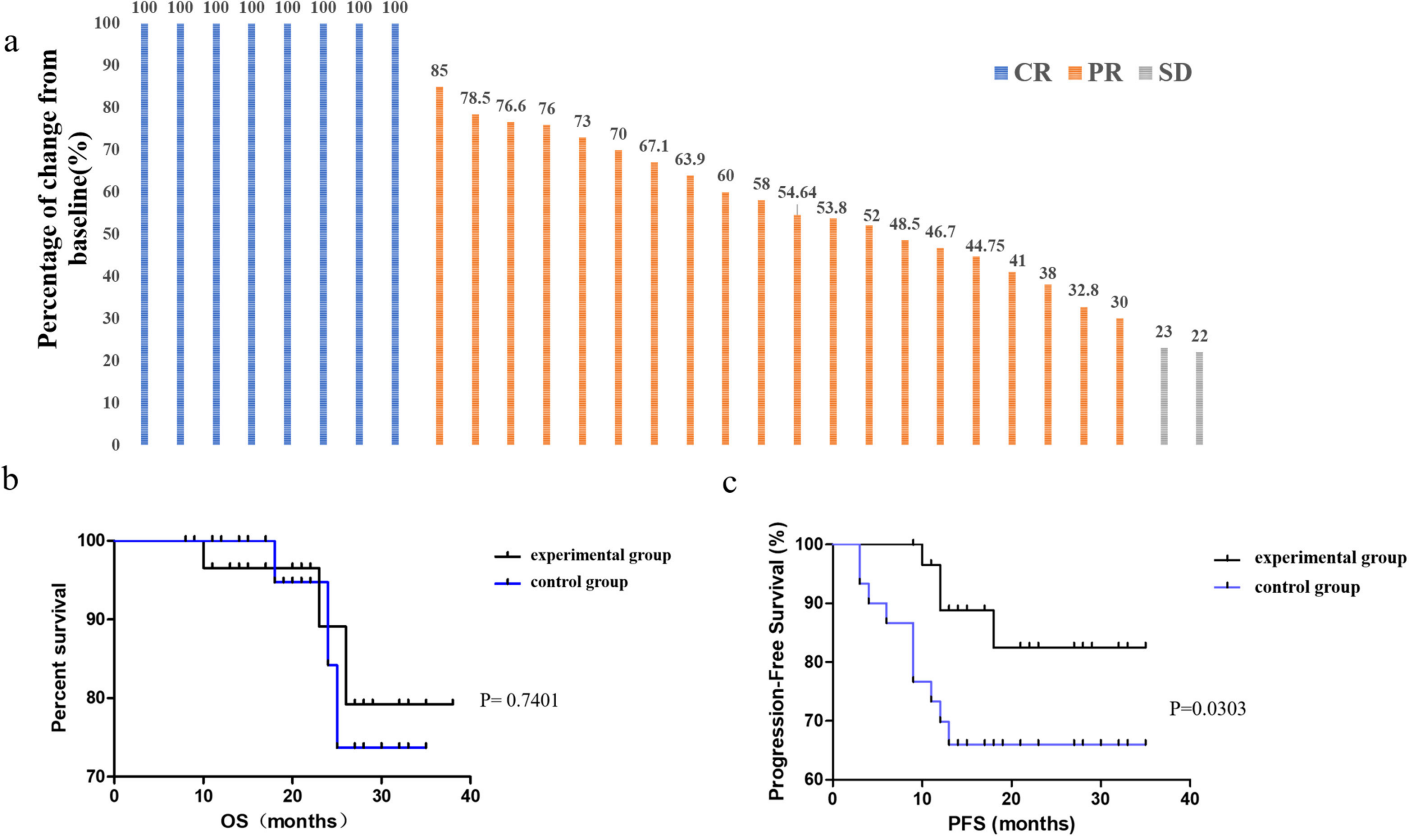
**(a)** Percent change in target lesion size from baseline after neoadjuvant chemoimmunotherapy (NACI) in the experimental group. **(b)** Kaplan–Meier curves comparing survival outcomes between the experimental and control groups. **(c)** Kaplan–Meier curves comparing progression-free survival between the experimental and control groups.

**Figure 2:**
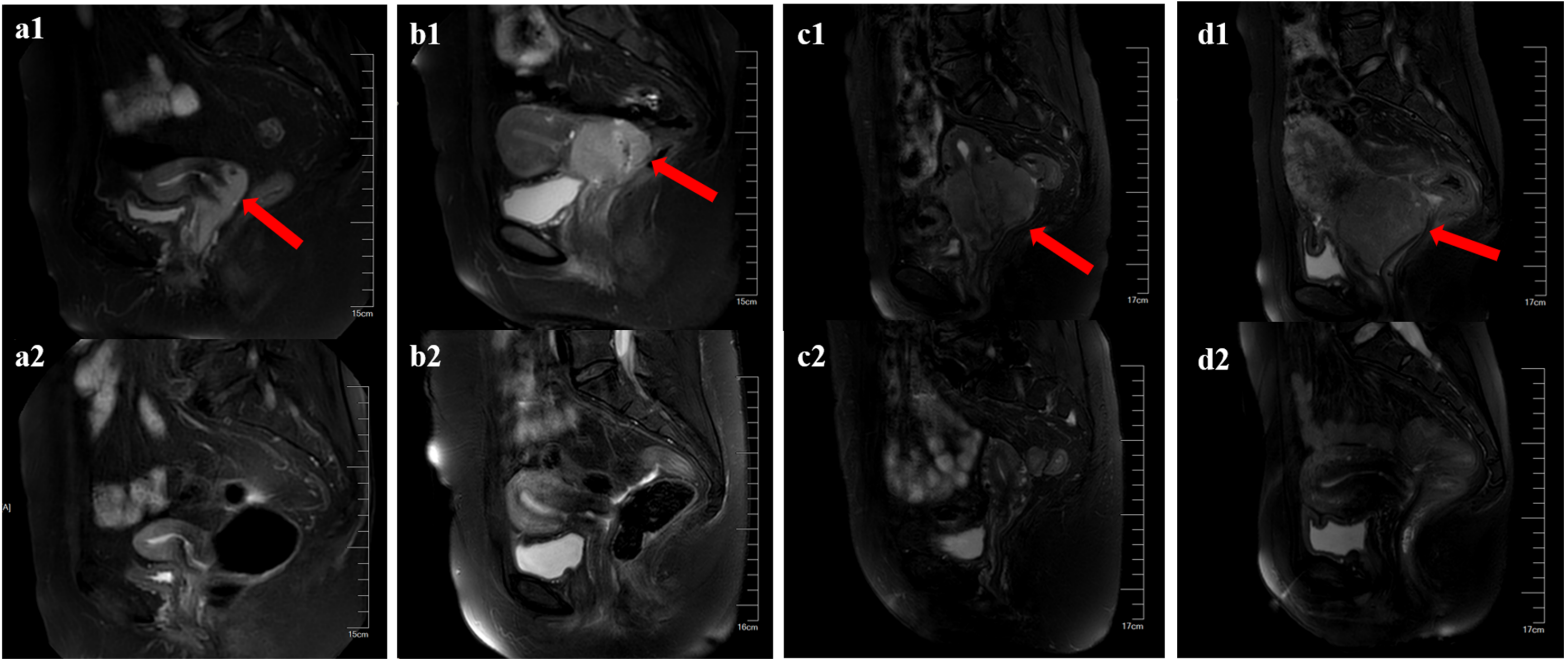
Evaluation of cervical lesions by magnetic resonance (MR) imaging before and after neoadjuvant chemoimmunotherapy in four patients from the experimental group, illustrating a complete response. **(a1, b1, c1, d1)** Pre-treatment contrast-enhanced T2-weighted (or T1-weighted) pelvic MR images (axial and sagittal views) from four patients in the experimental group, showing bulky, hyperintense (or enhancing) cervical masses (arrows) with well-defined local extent due to excellent soft-tissue contrast. **(a2, b2, c2, d2)** Corresponding post-treatment MR images for these four patients, respectively, confirm complete radiological resolution, as evidenced by stromal normalization and no residual abnormal signal or enhancement.

A total of 30 patients underwent the first efficacy evaluation after NACI and before radical radiotherapy (RR). The results showed 8 cases (26.7%) of complete response (CR) and 20 cases (66.7%) of partial response (PR), yielding an objective response rate (ORR) of 93.3% (**Figure 1a**). Notably, the highest CR rate (35.0%) was observed in patients who received two cycles of NACI (**Table 2**). **Figure 2** showcases the pre- and post-treatment MR (magnetic resonance) images for a subset of the experimental group, confirming complete response after neoadjuvant chemoimmunotherapy.

**Table 2:** Treatment response at different time points in the two groups.

Group / time point	N	CR (%)	PR (%)	ORR (%)
Experimental group				
After NACI (before RR)	30	8 (26.7)	20 (66.7)	28 (93.3)
By NACI cycles				
1 cycle	5	0 (0.0)	3 (60.0)	3 (60.0)
2 cycles	20	7 (35.0)	13 (65.0)	20 (100.0)
3 cycles	5	1 (20.0)	4 (80.0)	5 (100.0)
After NACI + RR	30	27 (90.0)	3 (10.0)	30 (100.0)
By FIGO stage				
IIIA	1	1 (100.0)	0 (0.0)	1 (100.0)
IIIB	8	7 (87.5)	1 (12.5)	8 (100.0)
IIIC	19	19 (100.0)	0 (0.0)	19 (100.0)
IVA	2	0 (0.0)	2 (100.0)	2 (100.0)
By histological type				
Squamous cell carcinoma	29	26 (89.7)	3 (10.3)	29 (100.0)
Adenocarcinoma	1	1 (100.0)	0 (0.0)	1 (100.0)
By baseline tumor size				
≤4 cm	2	2 (100.0)	0 (0.0)	2 (100.0)
>4 cm	28	25 (89.3)	3 (10.7)	28 (100.0)
By NACI cycles				
1 cycle	5	4 (80.0)	1 (20.0)	5 (100.0)
2 cycles	20	19 (95.0)	1 (5.0)	20 (100.0)
3 cycles	5	4 (80.0)	1 (20.0)	5 (100.0)
Control Group				
After RR	30	26 (86.7)	3 (10.0)	29 (96.7)

NACI, neoadjuvant chemoimmunotherapy; RR, radical radiotherapy; CR, complete response; PR, partial response; ORR, objective response rate (CR+PR).

#### 3.1.2 Final tumor response after NACI + RR in the experimental group

Following completion of NACI followed by radical radiotherapy (RR), the complete response (CR) rate in the experimental group increased to 90.0% (27/30), with a partial response (PR) rate of 10.0% (3/30), resulting in an objective response rate (ORR) of 100%. Subgroup analysis demonstrated an ORR of 100% across patients with different FIGO stages, histological types, number of NACI cycles, and baseline tumor sizes (**Table 2**).

#### 3.1.3 Final tumor response and comparison between groups

The final treatment response, assessed after the completion of all protocol-defined therapy (i.e., after NACI+RR in the experimental group and after RR in the control group), is summarized in **Table 2**. The objective response rate (ORR) was 100% (30/30) in the experimental group and 96.7% (29/30) in the control group. This difference was not statistically significant (P = 0.500, Fisher’s exact test). A numerically higher complete response (CR) rate was observed in the experimental group (90.0%, 27/30) compared to the control group (86.7%, 26/30); however, this descriptive difference was not statistically significant (P = 1.000, Fisher’s exact test).

Notably, a substantial objective response rate (ORR) of 93.3% (28/30) was achieved after NACI alone in the experimental group, before radiotherapy was initiated, which included 8 patients (26.7%) attaining a complete response at this early time point.

### 3.2 Survival analysis

#### 3.2.1 Univariate survival analysis

As of May 2025, the median follow-up was 20.5 months (range: 8–38 months). Neither median progression-free survival (PFS) nor overall survival (OS) had been reached. In the experimental group, 4 patients experienced recurrence or metastasis: 2 had local recurrence and 2 had distant metastasis. Three deaths occurred, two of which were tumor-related. In the control group, 10 patients had recurrence or metastasis, including 4 with local recurrence, 5 with distant metastasis, and 1 with both. Three deaths were due to tumor progression.

No significant difference in overall survival (OS) was observed between groups (log-rank test, P = 0.7401; HR = 0.945, 95% CI: 0.190-4.698). The 2-year OS rate was 89% in the experimental group versus 84% in controls (**Figure 1b**). The experimental group showed a trend toward improved progression-free survival (PFS) (log-rank test, P = 0.0303; HR = 0.364, 95% CI: 0.126-1.052), with 1-year PFS rates of 88.8% (experimental) versus 70.0% (control), and 2-year PFS rates of 82.5% versus 66.0%, respectively (**Figure 1c**).

#### 3.2.2 Multivariate analysis

The distribution of concurrent treatment regimens during RR is shown in **Table 1**. In the experimental group (n=30), 4 patients (13.3%) received RR alone, 5 (16.7%) received RR with concurrent immunotherapy, 11 (36.7%) received RR with concurrent chemotherapy, and 10 (33.3%) received RR with concurrent chemoimmunotherapy. In the control group (n=30), 2 patients (6.7%) received RR alone and 28 (93.3%) received RR with concurrent chemotherapy. No patients in the control group received RR with immunotherapy or RR with chemoimmunotherapy.

A multivariate Cox regression analysis was performed to adjust for potential confounders, including concurrent treatment during RR. As shown in **Table 3**, after adjusting for these variables, the association between NACI and improved PFS was not statistically significant by conventional criteria (adjusted HR = 0.367, 95% CI: 0.077–1.741, P = 0.209). Furthermore, none of the concurrent treatment regimens during RR (RR with chemotherapy, RR with immunotherapy, or RR with chemoimmunotherapy) were significantly associated with PFS compared with RR alone (all P > 0.05). The overall likelihood ratio test for the model was not significant (P = 0.285), indicating limited overall explanatory power, which is likely attributable to the small sample size and the low number of PFS events (n=14).

**Table 3:** Multivariate Cox regression analysis for progression-free survival.

Variable	β (coefficient)	SE	Wald χ²	P value	Hazard ratio (HR)	95% CI for HR
NACI (experimental vs. control)	-1.0029	0.7988	1.576	0.209	0.367	0.077–1.741
Concurrent treatment during RR (vs. RR alone)						
RR + chemotherapy	0.1635	1.0772	0.023	0.879	1.178	0.144–9.622
RR + immunotherapy	-11.8039	294.0078	0.002	0.968	0.000	7.69×10^255^ to 7.27×10^243^
RR + chemoimmunotherapy	0.4758	1.2979	0.134	0.714	1.609	0.128–20.220

The reference category for concurrent treatment is RR alone (radiotherapy without any concurrent systemic therapy).

Overall model likelihood ratio test: χ² = 5.021, df = 4, P = 0.285.

The extremely wide confidence interval for the “RR + immunotherapy” subgroup is due to very few or zero events in that category, leading to an unstable estimate. These results should be interpreted with caution given the limited sample size and low number of PFS events (n=14).

### 3.3 Safety

#### 3.3.1 Immune-related adverse events

In the experimental group, 16 patients (53.3%, 16/30) experienced a total of 17 immune-related adverse events (irAEs), with one patient experiencing two different types of irAEs. All events were grade 1 or 2 in severity. The most common irAEs were cardiac toxicity (5 cases) and endocrine toxicity (4 cases). No grade 3 or higher irAEs occurred (**Table 4**).

**Table 4:** Treatment-related adverse events in the experimental and control groups (N=60).

Type of adverse event	Experimental group (N=30)	Control group (N=30)	P-value
irAEs			
Cardiac	G1: 2; G2: 3	NA	NA
Endocrine	G1: 2; G2: 2	NA	NA
Pulmonary	G1: 2	NA	NA
Dermatological	G1: 2	NA	NA
Gastrointestinal	G1: 1	NA	NA
Hepatic	G1: 1	NA	NA
Neurological	G1: 1	NA	NA
Hematological	G1: 1	NA	NA
Total irAEs, n (%)	16 (53.3%) †	NA	NA
Radiotherapy-Related Adverse Events			
Intestinal	G1: 2; G2: 4	G1: 3; G2: 10; G3: 5	0.0033
Urinary	G2: 2	G1: 1; G2: 2	0.6120
Total radiotherapy-related AEs, n (%)	8 (26.7%)	21 (70.0%)	0.0017
Radiotherapy interruption, n (%)	4 (13.3%)	7 (23.3%)	0.5062

Toxicity was graded according to CTCAE v5.0: G1 (mild), G2 (moderate), and G3 (severe).

irAEs, immune-related adverse events; NA, not applicable.

Data are presented as number of patients (percentage).

Some patients experienced multiple adverse events, so percentages may not sum to 100%.

† The total number of irAE events was 17 among 16 patients in the experimental group, as one patient experienced two different types of irAEs.

#### 3.3.2 Radiotherapy-related adverse reactions

The incidence of intestinal adverse reactions related to radiotherapy was significantly lower in the experimental group than in the control group (20% vs. 60%, *P* = 0.0033). All intestinal reactions in the experimental group were grade 1-2 (6 cases, 20%), with no grade ≥3 events. In contrast, the control group had 13 cases (43.3%) of grade 1–2 and 5 cases (16.7%) of grade ≥3 intestinal reactions. There was no statistically significant difference in urinary adverse reactions between the two groups (*P* = 0.6120). Radiotherapy was interrupted due to adverse reactions in 4 cases (13.3%) in the experimental group and 7 cases (23.3%) in the control group, a difference which was not statistically significant (*P* = 0.5062) (**Table 4**).

## 4 Discussion

This study evaluated the efficacy and safety of a novel strategy combining neoadjuvant chemoimmunotherapy (NACI) with radical radiotherapy (RR) in patients with locally advanced cervical cancer. The experimental group demonstrated promising short-term efficacy: the objective response rate (ORR) was 93.3% after NACI and reached 100% upon completion of the combined treatment (NACI followed by RR). The complete response (CR) rate was 90.0%, which was higher than that in the control group, although the difference was not statistically significant. After a median follow-up of 20.5 months, the 2-year progression-free survival (PFS) rate was significantly higher in the experimental group (82.5% vs. 66.0% in the control group; hazard ratio [HR] = 0.364, P = 0.0303). It should be noted, however, that the relatively small sample size limits the statistical power and generalizability of these findings. Immune-related adverse events (irAEs) were observed in 53.3% of patients in the experimental group, with the majority being grade 1-2. Importantly, the incidence of grade ≥3 radiotherapy-related gastrointestinal toxicity was significantly lower in the experimental group (0% vs. 16.7%, P = 0.0033). While these preliminary results indicate potential benefits of the combined modality, further validation in larger prospective studies is warranted to confirm the efficacy and safety profile of this regimen.

Since the late 1990s, concurrent chemoradiotherapy (CCRT) has been established as the standard treatment for locally advanced cervical cancer based on multiple large-scale trials (10–16). However, patients with large tumor volumes or hypoxic regions often have residual disease after CCRT, which compromises local control and adversely affects long-term survival. To address this limitation, ongoing research aims to develop more effective CCRT-based strategies to improve outcomes. Two recent phase III trials, INTERLACE and KEYNOTE-A18, have shown promising results (17–19). Neoadjuvant chemotherapy (NACT) can reduce tumor burden, decrease lymphovascular invasion, and increase radiosensitivity. The precursor CxII study (20) to INTERLACE found that NACT with paclitaxel and carboplatin before definitive CRT significantly improved response rates, with an objective response rate (ORR) of 70% after NACT rising to 85% after CRT. The INTERLACE trial (18) further showed that induction chemotherapy followed by CCRT significantly improved survival, with 5-year PFS of 72% vs. 64% (p = 0.013) and 5-year OS of 80% vs. 72% (p = 0.015). Based on these findings, the 2025 NCCN guidelines now recommend induction chemotherapy for select patients. In parallel, the KEYNOTE-A18 trial evaluated the addition of concurrent and adjuvant pembrolizumab to CCRT (19). The pembrolizumab group had an ORR of 79% versus 76% with placebo. At a median follow-up of 29.9 months, median OS was not reached in either group, but the 3-year OS was 82.6% with pembrolizumab compared to 74.8% with placebo, leading to its FDA approval for stage III-IVA disease (21, 22). The KEYNOTE-A18 trial established pembrolizumab plus concurrent chemoradiotherapy (CCRT) followed by adjuvant pembrolizumab as a new standard for high risk locally advanced cervical cancer (LACC). Beyond survival benefits, recent patient−reported outcome data from the KEYNOTE−A18 trial (23) confirmed that adding pembrolizumab to CCRT did not negatively affect health−related quality of life, supporting the tolerability and patient−centered value of this regimen. A recent Bayesian network meta−analysis (24) of phase 3 RCTs confirmed that, among immune checkpoint inhibitors, pembrolizumab plus chemoradiotherapy ranks highest for overall and progression−free survival in locally advanced cervical cancer, outperforming durvalumab.

Considering the recently reported KEYNOTE−A18 trial, and acknowledging that both regimens include immunotherapy, it might be worthwhile to discuss some possible comparative aspects of our NACI−RR regimen that could be considered advantageous, especially regarding efficacy, safety, and clinical applicability. From an efficacy perspective, our NACI−RR regimen was associated with an ORR of 100% after radiotherapy and a 2−year PFS of 82.5% in this limited cohort. Cross−trial comparisons are inherently problematic, and any indirect comparison with the KEYNOTE−A18 trial should be regarded as exploratory. With that in mind, the 2−year PFS in our experimental group might be broadly comparable to that observed in the KEYNOTE−A18 pembrolizumab arm (approximately 70−75%), though such a comparison warrants considerable caution. The CR rate in our study was 90.0%, which appears numerically higher than the ORR of 79% reported in KEYNOTE−A18, but this difference could be influenced by multiple factors, including patient selection and assessment timing. One might hypothesize that the distinct treatment sequence — neoadjuvant chemoimmunotherapy before radiotherapy versus concurrent/adjuvant immunotherapy alone — could potentially contribute to deeper tumor debulking and earlier immune activation, but this remains speculative and requires validation in future prospective studies. Cross−trial comparisons of safety and clinical applicability are difficult. Unlike KEYNOTE−A18 (75% grade ≥3 AEs; 4.7% grade ≥3 irAEs), our study had no grade ≥3 irAEs, and grade ≥3 intestinal toxicity was 0% (vs. 16.7% in controls). NACI−induced tumor shrinkage might reduce intestinal radiation exposure, but this requires validation. Any toxicity advantage of the induction−first approach remains preliminary. These findings are preliminary and require prospective validation.

Thus, INTERLACE established the benefit of NACT before CCRT, while KEYNOTE-A18 showed the value of adding immunotherapy during and after CCRT. Additionally, a study by Liu et al. (6) reported a pathological complete response rate of 32.6% with neoadjuvant chemoimmunotherapy alone in stage IB3/IIA2 patients. Jyoti Mayadev’s research (25) compared neoadjuvant versus concurrent atezolizumab with CCRT and found higher ORR after 28 days with neoadjuvant treatment (69% vs. 40%), suggesting potential benefit even from a short course of immune induction. In summary, current evidence suggests that immune-chemo combinations may represent a promising induction therapy strategy for cervical cancer, though further validation in larger prospective studies is still needed to confirm their efficacy and safety. Recent meta−analytic evidence further supports that platinum−paclitaxel induction chemotherapy ≤6 weeks followed by chemoradiotherapy improves survival in locally advanced cervical cancer (26).

Our study investigated a treatment strategy involving immunotherapy combined with chemotherapy as induction, followed by radical radiotherapy, in patients with stage IIIA to IVA (FIGO 2018) locally advanced cervical cancer with a focus on those presenting with a maximum tumor diameter ≥5 cm or lymph node metastasis. The results showed an objective response rate (ORR) of 93.3% after induction therapy, increasing to 100% following subsequent radiotherapy. This compares favorably with historical data: previous studies have reported ORRs of up to 85.7% with cisplatin plus docetaxel-based concurrent chemoradiotherapy (CCRT) (27), while neoadjuvant chemotherapy (NACT) with paclitaxel and carboplatin followed by CRT yielded ORRs of 70% after NACT and 85% after CRT (20). Although the ORR in our study appears numerically higher than these benchmarks and that of our control group, the relatively small sample size necessitates cautious interpretation of these comparisons. With a median follow-up of 20.5 months, median progression-free survival (PFS) and overall survival (OS) had not yet been reached in the experimental group. PFS was significantly improved compared with the control group, suggesting the possibility of long-term survival benefits. However, these promising preliminary findings require further validation in larger cohorts. In conclusion, the strategy of immune-chemo induction prior to radical radiotherapy was associated with encouraging response and survival outcomes, indicating a potentially promising therapeutic avenue that merits further investigation.

In the experimental group, patients who received two cycles of NACI achieved the highest complete response (CR) rates: 35% after NACI alone, rising to 95% after the combined NACI and RR. This pattern suggests that two cycles may represent an optimal treatment duration. In contrast, a single cycle appeared suboptimal, whereas extending to three cycles did not yield a markedly superior benefit. Furthermore, the regimen showed notable efficacy in patients with larger tumors (>4 cm), among whom 89.3% still achieved a CR. This finding implies potential effectiveness even in this typically less favorable prognostic subgroup, offering a promising alternative therapeutic option. The observed outcomes in the experimental group might be partly explained by a possible synergistic effect between immunotherapy and chemotherapy (28, 29), which could enhance initial tumor burden reduction. This may allow subsequent radiotherapy to act more effectively on a smaller residual tumor volume at standard doses. Additionally, the tri-modality approach (immunotherapy, chemotherapy, and radiotherapy) may collaboratively modulate the immunosuppressive tumor microenvironment: chemoradiation can promote tumor antigen release, while concurrent immunotherapy may potentiate immune activation and sustain antitumor responses.

Although therapeutic efficacy is paramount, managing radiotherapy-related side effects remains a significant clinical challenge in cervical cancer treatment. In recent years, advanced techniques such as IMRT and IGABT have been increasingly employed, allowing for more targeted irradiation and reduced radiation exposure to organs such as the intestines and bladder. However, completely avoiding intestinal and urinary toxicities during radiotherapy remains difficult.

During pelvic irradiation, up to 84% of patients may experience some form of acute radiotherapy toxicity (30–32). Long-term follow-up indicates that over 50% of patients may develop chronic intestinal toxicities within five years after external beam radiotherapy, including symptoms such as chronic diarrhea, constipation, and rectal bleeding (33). The reported incidence rates of grade 3–4 chronic gastrointestinal and genitourinary toxicities are approximately 4.1% and 2.7%, respectively, while severe complications such as rectovesical or rectovaginal fistula occur in about 1.4% of cases (34). Radiation dose to the rectum and bladder is a known influencing factor for such toxicities (35). Furthermore, substantial tumor shrinkage—with volume reductions of 60%–80% reported—can occur during external beam radiotherapy (33, 36). As the tumor regresses, organs at risk (OARs) may shift into the treatment field, potentially receiving unintended higher radiation doses, a phenomenon observed more frequently in locally advanced cervical cancer.

In this study, the incidences of radiotherapy-related intestinal and urinary toxicities in the experimental group were 20% and 3.3%, respectively. The intestinal toxicity rate was significantly lower than that in the control group (60%) and also appeared lower than the ranges commonly reported for concurrent chemoradiotherapy (CCRT) alone (intestinal toxicity: 40.9%–68.6%; urinary toxicity: 12.5%–26%) (15, 16). We suggested that tumor volume reduction following NACI induction therapy might enable a reduction in planning target volume (PTV) margins, thereby decreasing radiation exposure to OARs and potentially reducing the risk of radiotherapy-related toxicity. Although a higher incidence of irAEs was noted in the experimental group, most were manageable, with no grade 3 or higher irAEs observed. Moreover, irAEs tend to be transient, whereas radiotherapy-induced toxicities are more likely to be persistent or irreversible.

In this study, treatment response was assessed using imaging studies combined with clinical gynecologic examination. Gynecological examination plays a crucial role in cervical cancer staging. Prior to treatment, it helps characterize baseline tumor features (e.g., size, texture, and extent of invasion). Following treatment, it allows for evaluation of tumor regression, changes in consistency, and possible involvement of parametrial and paravaginal tissues. Comparing pre- and post-treatment findings supports a comprehensive outcome assessment. Regarding the timing of assessment, in contrast to the INTERLACE and KEYNOTE-A18 trials, where responses were evaluated 12 weeks post-radiotherapy, we performed evaluations at 1 month after radiotherapy completion. This earlier time point was selected because the acute tissue reactions from radiotherapy often subside by this stage, allowing the initial treatment response to be more clearly assessed. This approach may enable earlier identification of residual disease, which could facilitate timely initiation of salvage therapy if indicated.

Although the unadjusted analysis showed a significant PFS benefit in the experimental group (HR = 0.364, P = 0.0303), this association became non−significant after adjusting for concurrent radiotherapy regimens (adjusted HR = 0.367, P = 0.209). The minimal change in HR suggests that heterogeneity in radiotherapy regimens did not meaningfully confound the estimate, and the loss of significance is likely due to reduced statistical power from the limited number of events. However, several factors caution against overinterpreting this negative multivariate result. First, the analysis was limited by small sample size (30 per group) and few PFS events (n = 14), leading to low statistical power and increased risk of type II error (failing to detect a true effect). Importantly, the adjusted HR (0.367) is nearly identical to the unadjusted HR (0.364), indicating that adjustment did not meaningfully reduce the estimated benefit; the loss of significance likely reflects insufficient power. Second, the overall model was non−significant (likelihood ratio test, P = 0.285), further confirming limited explanatory power due to the low event count. Thus, although the multivariate analysis did not identify an independent effect of NACI on PFS that reached conventional statistical significance, a benefit cannot be ruled out. The point estimate of the HR remained consistent, and a significant reduction in radiotherapy−related intestinal toxicity was observed (20% vs. 60%, P = 0.0033). These findings might be compatible with a clinically meaningful advantage of the NACI−RR regimen, although confirmation in larger, adequately powered studies would be desirable.

Limitations and Future Directions

Given the retrospective nature of this study, the sample size was not determined *a priori*. To support the statistical conclusions, we performed a post−hoc power calculation using the observed 2−year progression−free survival (PFS) rates (experimental group: 82.5%, control group: 66.0%) with a two−sided significance level of 0.05. The calculated power for detecting this difference was approximately 62% with the current sample size of 30 patients per group, indicating a moderate risk of type II error and that the relatively small sample size may increase the risk of false−negative or overestimated effect sizes. Therefore, the results should be interpreted with caution, and larger prospective studies are warranted to confirm the findings.

This study has several limitations. First, it is a single-center retrospective analysis with a relatively small sample size, and the survival outcomes require validation through longer follow-up. Second, because the control group did not receive induction chemotherapy, a potential contribution from chemotherapy alone during the induction phase cannot be entirely excluded. Third, the use of different immunotherapeutic agents in the experimental group may have introduced heterogeneity. Fourth, the lack of comprehensive biomarker data (e.g., PD-L1 expression, tumor mutational burden, or tumor-infiltrating lymphocytes) hampers a molecular-level interpretation of the observed efficacy differences. Fifth, due to only 14 PFS events, the multivariate analysis was limited in statistical power. With such a small number of events, Cox models may be prone to overfitting. Therefore, the non−significant finding for NACI (P = 0.209) cannot be interpreted as evidence of no effect, and the wide confidence interval (0.077–1.741) suggests that larger studies may be warranted. Future trials could consider standardizing or stratifying by concurrent radiotherapy regimens to reduce potential confounding.

## 5 Conclusion

Based on preliminary real-world data, this study provides exploratory clinical observations on the “neoadjuvant immunochemotherapy followed by radical radiotherapy” regimen for locally advanced cervical cancer. The results suggest potential short-term efficacy and a potential progression-free survival benefit, with a generally acceptable profile of immune-related adverse events. A substantial reduction in radiotherapy-related intestinal toxicity to organs at risk was also observed, contributing to a tolerable safety profile. These preliminary findings warrant further investigation in larger-scale studies.

Multicenter, randomized, phase III trials are required to confirm the long-term survival outcomes and to define more clearly the optimal number of neoadjuvant immunochemotherapy (NACI) cycles and the duration of subsequent immune maintenance therapy.

## Data Availability

The original contributions presented in the study are included in the article/supplementary material. Further inquiries can be directed to the corresponding author.
